# G-Alpha Subunit Abundance and Activity Differentially Regulate β-Catenin Signaling

**DOI:** 10.1128/MCB.00422-18

**Published:** 2019-02-15

**Authors:** Arshiya Banu, Karen J. Liu, Alistair J. Lax, Agamemnon E. Grigoriadis

**Affiliations:** a Department of Microbiology, King’s College London, Guy’s Hospital, London, United Kingdom; b Centre for Craniofacial and Regenerative Biology, King’s College London, Guy’s Hospital, London, United Kingdom

**Keywords:** β-catenin, G-alpha subunits, G-proteins, *Pasteurella multocida* toxin

## Abstract

Heterotrimeric G proteins are signal transduction proteins involved in regulating numerous signaling events. In particular, previous studies have demonstrated a role for G-proteins in regulating β-catenin signaling. However, the link between G-proteins and β-catenin signaling is controversial and appears to depend on G-protein specificity. We describe a detailed analysis of a link between specific G-alpha subunits and β-catenin using G-alpha subunit genetic knockout and knockdown approaches. The Pasteurella multocida toxin was utilized as a unique tool to activate G-proteins, with LiCl treatment serving as a β-catenin signaling agonist. The results show that Pasteurella multocida toxin (PMT) significantly enhanced LiCl-induced active β-catenin levels in HEK293T cells and mouse embryo fibroblasts. Evaluation of the effect of specific G-alpha proteins on the regulation of β-catenin showed that G_q/11_ and G_12/13_ knockout cells had significantly higher levels of active and total β-catenin than wild-type cells. The stimulation of active β-catenin by PMT and LiCl was lost upon both constitutive and transient knockdown of G_12_ and G_13_ but not G_q_. Based on our results, we conclude that endogenous G-alpha proteins are negative regulators of active β-catenin; however, PMT-activated G-alpha subunits positively regulate LiCl-induced β-catenin expression in a G_12/13_-dependent manner. Hence, G-alpha subunit regulation of β-catenin is context dependent.

## Abstract (precis)

Heterotrimeric G proteins are signal transduction proteins involved in regulating numerous signaling events. In particular, previous studies have demonstrated a role for G-proteins in regulating β-catenin signaling.

## INTRODUCTION

The heterotrimeric G proteins represented by the G_s_, G_i/o_, G_q/11_, and G_12/13_ families serve as essential links between the large number of G-protein-coupled receptors (GPCRs) that respond to many agonists and the activation of several defined intracellular signaling pathways (1–3). Each G-protein family is characterized based on specific alpha subunits and is classically associated with a specific signaling pathway. Thus, G_s_ stimulation activates adenylate cyclase, whereas G_i_ stimulation inhibits adenylate cyclase activity (4). Activation of G_q/11_ stimulates phospholipase C (PLC) and subsequently protein kinase C and calcium-linked signaling (5, 6), whereas the activation of the G_12/13_ family promotes the activity of Rho and cytoskeleton rearrangements (7–11). Although each of the G-protein families is associated with specific signaling activation, there is some evidence demonstrating the interregulation of G-alpha subunits and cross-activation of signaling pathways. For instance, G_q_, which stimulates PLC, can also activate Rho signaling proteins, which are classically assigned to G_12/13_ signaling (12–16). The levels of G-alpha subunits have also been shown to have some degree of interregulation. For example, the short interfering RNA (siRNA) knockdown of G_q_ resulted in an upregulation of G_i_ subunits, leading to an activation of G_i_-mediated signaling events (17). As well as this interaction among G-protein signaling pathways, G-proteins also impinge on other signaling pathways. In particular, G-proteins are known to interact with and regulate the β-catenin signaling pathway.

β-Catenin is a multifunctional protein that can exhibit cell membrane, cytoplasmic, and nuclear localization to interact with a multitude of signaling cascades and transcription factors (18–20). Interactions between β-catenin and G-proteins have been studied largely in the context of canonical Wnt signaling, an evolutionarily conserved pathway which involves the translocation of β-catenin into the nucleus, where it activates gene transcription (21). In the absence of Wnt ligands, the level of cytoplasmic β-catenin is regulated by the phosphorylation, ubiquitination, and proteosomal degradation mediated by a destruction complex consisting of axin, adenomatous polyposis coli (APC), and glycogen synthase kinase 3 (GSK3) (21–25). Studies on the cross talk between G-proteins and Wnt/β-catenin signaling have revealed complex interactions. Activation of β-catenin signaling following stimulation of the canonical Wnt/Frizzled pathway has been shown to be dependent in part on G_q_ through inhibition of GSK3β, suggesting that some G-alpha subunits positively regulate the canonical Wnt pathway (26–29). Meigs et al. reported that in cells lacking APC, β-catenin-mediated transcriptional activation is upregulated by expression of activated G_12_ or G_13_ (30). G_o_, a member of the G_i/o_ family, interacts with the Wnt signaling mediator Dishevelled and plays an essential role in Wnt3a-mediated activation of the Jun N-terminal kinase (31–34). In contrast to the findings described above, studies on fibrous dysplasia showed that activated G_q_, G_11_, G_12_, and G_13_ proteins had no significant roles in regulating β-catenin, while only activated G_s_ was shown to stimulate the Wnt signaling pathway (35). In the broader view of β-catenin signaling independent of Wnt signaling, these studies indicate that the abilities of specific G-alpha subunits to regulate β-catenin signaling are variable and context dependent. Indeed, G-protein and β-catenin signaling cross talk has often been studied by considering each individual G-alpha subunit in isolation. However, as levels of one G-protein family are known to affect the expression and function of other G-protein families, the interrelation between these pathways could be quite complex. Moreover, the role of endogenously activated G-proteins in β-catenin signaling in the absence of exogenous ligand stimulation is poorly understood.

In this work, we have investigated the role of basal and activated G_q/11_ and G_12/13_ families in the regulation of active β-catenin. In this regard, the Pasteurella multocida toxin (PMT) provides a novel tool to dissect these pathways. PMT is a potent intracellularly acting toxin which activates three families of heterotrimeric G-proteins: G_q/11,_ G_12/13_, and G_i/o_ (36–41). PMT acts to deamidate a key glutamine (Q) to glutamic acid (E) in the target G-alpha subunits involved in GTP hydrolysis, leading to chronically activated G-protein function (41–43); these PMT-modified G-alpha subunits can be detected specifically using an anti-QE antibody that recognizes PMT-modified G-alpha subunits (44). As PMT treatment stimulates the activation of various G-protein-mediated downstream events, it offers a unique opportunity to explore the differences between the effects of basal and activated G-proteins on β-catenin signaling in the absence of exogenous stimulation. We have investigated in both fibroblastic and epithelial cell model systems whether PMT treatment leads to an activation of β-catenin signaling and, further, which G-protein families, if any, are likely to be responsible. In this report, we have used the GSK3 inhibitor LiCl as a direct activator of β-catenin signaling independently of the Wnt pathway to focus on how differential activation of specific G-alpha protein affects active β-catenin expression. Short-term activation of G-proteins using PMT enhanced the LiCl-induced active β-catenin levels in a G_12/13_-dependent manner. In contrast, cells with genetic inactivation of G_q/11_ and G_12/13_ showed upregulated β-catenin signaling. Together, our findings suggest that the link between G-alpha subunits and β-catenin signaling is purely dependent on G-protein abundance and activity.

## RESULTS

### PMT activates G-alpha subunits and enhances LiCl-induced β-catenin signaling.

**FIG 1:**
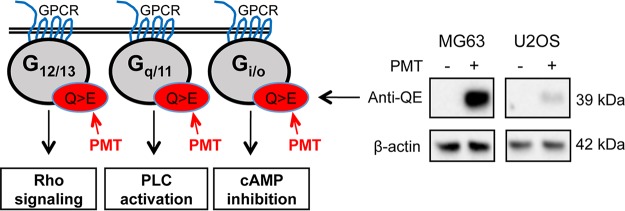
PMT activates G-alpha subunits. (A) Schematic representation of general G-protein signaling and downstream signaling pathways showing PMT activation of G-alpha subunits by specific glutamine deamidation (Q > E) (red). Western blot analysis of MG63 and U2OS cells left unstimulated or stimulated with PMT (40 ng/ml, 24 h) confirms the PMT modification of G-alpha subunits using a specific anti-QE antibody. A similar induction was observed in HEK293T cells (not shown). β-Actin expression was used as a loading control.

**FIG 2:**
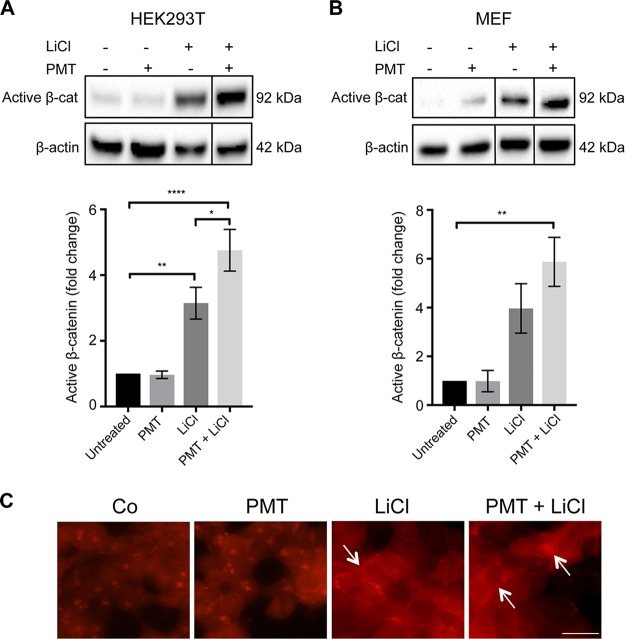
PMT enhances LiCl-induced β-catenin signaling. (A and B) Western blot analysis of active β-catenin levels in HEK293T cells (A) and wild-type MEFs (B) treated with PMT (HEK, 40 ng/ml; MEF, 20 ng/ml), LiCl (25 mM), either alone or together, or left untreated for 24 h. Levels of active β-catenin (Active β-cat) were evaluated using a specific β-catenin antibody on whole-cell extracts. β-Actin was used as a loading control. The histograms represent densitometric analysis of pooled data from three independent experiments normalized to their respective untreated control cells. The data represent the means ± standard errors of the means (SEM). Statistical significance was determined by Tukey’s test (*n* = 3): *P* < 0.05 (*), *P* < 0.001 (**), and *P* < 0.0001 (****). (C) Immunofluorescence analysis of active β-catenin expression. Representative images of HEK293T cells treated with PMT (40 ng/ml) and LiCl (25 mM), either alone or in combination for 24 h or left untreated (Co). The cells were stained with an antibody specific for active β-catenin, showing membrane-associated and nuclear staining (arrows) and imaged as described in Materials and Methods. Scale bar, 20 μm.

We used PMT to dissect the role of G-proteins in the regulation of active β-catenin. Western blotting using an anti-QE antibody (44) confirmed that PMT treatment caused the expected Q > E modification in G-alpha proteins (Fig. 1). The effect of PMT on β-catenin signaling was evaluated in HEK293T cells and wild-type mouse embryonic fibroblasts (MEFs) by Western blotting using a specific antibody against nonphosphorylated β-catenin, referred to here as active β-catenin. LiCl, a classical GSK inhibitor, was used to activate β-catenin signaling (45, 46). PMT treatment alone did not induce significant changes in the levels of active β-catenin in either HEK293T cells or wild-type MEFs. In contrast, PMT was shown to significantly enhance the LiCl-induced stimulation of active β-catenin after 24 h (Fig. 2A and B) but not 6 h (data not shown). Immunofluorescence staining confirmed the increase in active β-catenin expression in cells treated with both PMT and LiCl, including distinct membrane-associated expression of β-catenin, indicating engagement with cadherins (Fig. 2C). The synergy with LiCl stimulation of β-catenin suggests that PMT-modified G-proteins further regulate the levels of active β-catenin by interacting with components of the β-catenin destruction complex.

### G-proteins negatively regulate the levels of active β-catenin.

**FIG 3:**
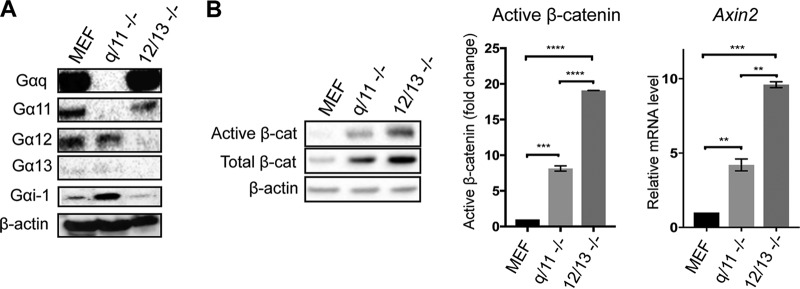
G_q/11_^−/−^ and G_12/13_^−/−^ cells show upregulated β-catenin signaling. (A) Characterization of G_q/11_^−/−^ and G_12/13_^−/−^ cells using Western blotting. Whole-cell extracts were prepared from wild-type (MEF), G_q/11_^−/−^ (q/11 -/-), and G_12/13_^−/−^ (12/13 -/-) MEFs. Levels of specific G-alpha subunits were evaluated using specific antibodies as indicated. Representative β-actin expression was used as a loading control. (B) Western blot analysis of active β-catenin (Active β-cat) and total β-catenin (Total β-cat) content in MEF, q/11^−/−^, and 12/13^−/−^ MEFs. The histograms represent densitometric analysis of pooled data from three independent experiments for active β-catenin and two independent experiments for qPCR analysis of axin2 expression compared to that of the wild-type MEFs, as indicated. The respective band intensities for Western blotting and fold change values for qPCR analysis were normalized to the respective β-actin levels and to untreated controls. The data represent the means ± SEM. Statistical significance was determined by Tukey’s test: **, *P* < 0.01; ***, *P* < 0.001; and ****, *P* < 0.0001.

Since PMT was found to be a potent activator of G-proteins and enhanced the levels of LiCl-induced active β-catenin, we next investigated the role of specific G-alpha subunits by using MEFs depleted for either G_q/11_ or G_12/13_ (47). Western blotting using specific G-alpha subunit antibodies confirmed the absence of specific G-alpha proteins in the relevant G_q/11_^−/−^ and G_12/13_^−/−^ MEFs (Fig. 3A). Interestingly, basal levels of G_i-1_ were upregulated ∼2-fold in G_q/11_^−/−^ but not in G_12/13_^−/−^ MEFs compared to those of wild-type cells (Fig. 3A). This suggests that G_q/11_ is a negative regulator of G_i-1_, consistent with our previous observations (42). The levels of G_s_ were not different among these three cell lines (data not shown).

Measurement of β-catenin in these three cell lines showed that cells lacking G_q/11_ or G_12/13_ subunits had ∼8-fold or 20-fold higher levels of active β-catenin, respectively, than wild-type MEFs (Fig. 3B). Interestingly, increases in basal total β-catenin levels were also observed (Fig. 3B). Quantitative PCR (qPCR) analysis of *Axin2* gene expression provided a readout of active β-catenin-dependent transcription and showed that G_q/11_^−/−^ MEFs had ∼5-fold and G_12/13_^−/−^ MEFs had ∼10-fold upregulated expression of *Axin2* compared to that of the wild-type MEFs, correlating well with the changes in active β-catenin levels (Fig. 3B). These results demonstrate that endogenous levels of G_q/11_ and G_12/13_ negatively regulate β-catenin signaling and that G_12/13_ appears to inhibit the pathway significantly more than G_q/11_.

### PMT enhancement of β-catenin expression is lost in G_q/11_ and G_12/13_ knockout cells.

**FIG 4:**
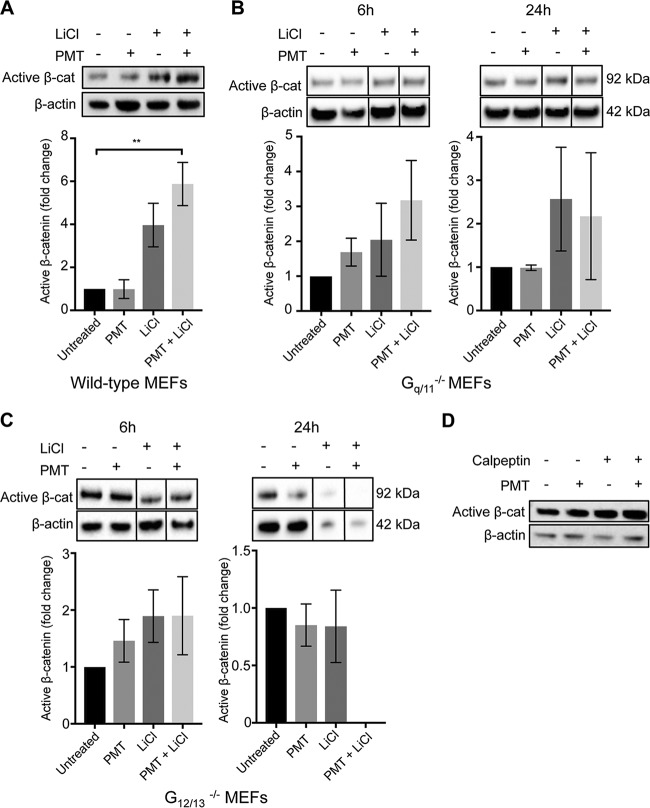
PMT enhancement of active β-catenin expression is lost in G_q/11_^−/−^ and G_12/13_^−/−^ cells. (A to C) Western blot analysis of active β-catenin expression in wild-type (A), G_q/11_^−/−^ (B), and G_12/13_^−/−^ (C) MEFs. Cells were stimulated with PMT (20 ng/ml) and LiCl (25 mM), either alone or in combination, or left untreated for 6 h or 24 h as indicated. Levels of active β-catenin (Active β-cat) were evaluated using a specific β-catenin antibody on whole-cell extracts. β-Actin was used as a loading control. The histograms represent densitometric analysis of pooled data from three independent experiments. The data represent the means ± SEM. *n* = 3. *, *P* < 0.05 by Tukey’s test. (C) Treatment of G_12/13_^−/−^ cells with both PMT and LiCl showed cytotoxicity and the quantification of active β-catenin was not possible. (D) Inhibition of calpain does not affect the PMT induction of active β-catenin. Western blot analysis of active β-catenin levels in HEK293T cells treated with PMT (40 ng/ml) and calpeptin (15 μM), either alone or in combination, or left untreated for 6 h, as indicated. Active β-catenin and β-actin were evaluated using specific antibodies described for panels A to C. The data are representative of three independent experiments.

As PMT enhanced the LiCl-induced active β-catenin levels, we next investigated if this was dependent on a specific G-alpha protein family. We stimulated the G-alpha-deficient cells with PMT and LiCl and quantified the levels of active β-catenin by Western blotting. Τhe results showed that in G_q/11_^−/−^ MEFs, neither PMT nor LiCl treatment alone or in combination for 6 or 24 h caused a significant stimulation of active β-catenin compared to that of the untreated cells or wild-type MEFs (Fig. 4A and B). This did not appear to be due to indirect effects of intracellular calpain, which is an established G_q_ effector that inhibits β-catenin (48), as treatment with the calpain inhibitor, calpeptin, had no effects on PMT-stimulated β-catenin (Fig. 4D).

Cells lacking G_12/13_ were more severely affected. In G_12/13_^−/−^ cells, PMT did not affect the levels of active β-catenin after 6 h, and after 24 h the levels of active β-catenin were severely compromised, being significantly decreased compared to those of the untreated cells (Fig. 4C). LiCl treatment alone did not alter the levels of active β-catenin at 6 h, but after 24 h some cytotoxicity was seen (data not shown). Combined treatment of G_12/13_^−/−^ MEFs with PMT and LiCl also resulted in further (∼90%) cell cytotoxicity (data not shown). Since both G_q/11_^−/−^ and, in particular, G_12/13_^−/−^ null cells have high endogenous levels of active β-catenin (Fig. 3B), it is possible that any further induction of active β-catenin using an agonist would compromise cell viability. Nevertheless, these results suggest that the PMT enhancement of LiCl-induced β-catenin is dependent on its ability to activate G-alpha subunits.

### PMT-enhanced active β-catenin is G_12/13_ dependent.

**FIG 5:**
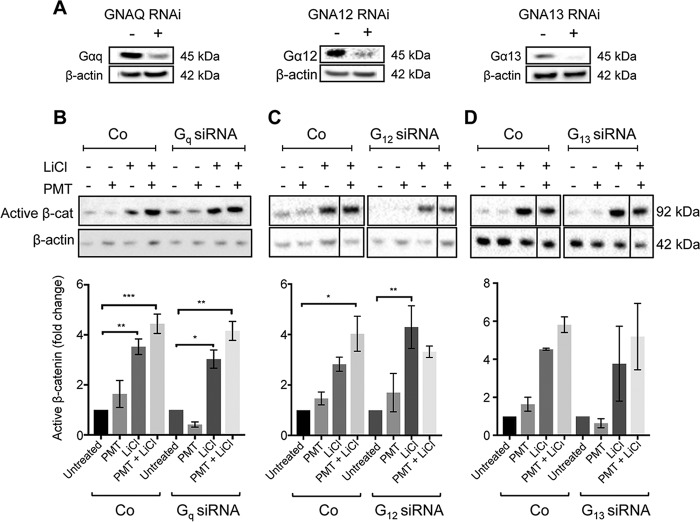
G-alpha subunit knockdown affects PMT and LiCl induction of active β-catenin. (A) Western blot analysis of G-alpha subunit protein expression in cells with the indicated G-protein knockdown. HEK293T cells were transfected with a specific siRNA against GNAQ (Gαq), GNA12 (Gα12), and GNA13 (Gα13) or with a nontargeting control, as described in Materials and Methods. The knockdown efficiency of each siRNA was measured using specific G-alpha subunit antibodies on whole-cell extracts. β-Actin was used as a loading control. (B to D) Western blot analysis of active β-catenin expression in G_q_ (B), G_12_ (C), and G_13_ (D) knockdown HEK293T cells. Cells were transiently transfected with either siRNA against the indicated G-alpha subunits or with nontargeting oligonucleotides (Co). Transfected cells were treated with PMT (40 ng/ml) and LiCl (25 mM), either alone or in combination, or left untreated for 24 h. Levels of active β-catenin (Active β-cat) were evaluated using a specific β-catenin antibody on whole-cell extracts. β-Actin was used as a loading control. Band intensity of active β-catenin was normalized against its respective β-actin. The fold changes were generated for each group compared to the respective untreated controls. The histograms represent densitometric analysis of pooled data from three independent experiments. The data represent the means ± SEM. Statistical significance was determined by Tukey’s test (*n* = 3): *, *P* < 0.05; **, *P* < 0.01; ***, *P* < 0.001; and ****, *P* < 0.0001.

The dramatic differences in PMT- and LiCl-regulated β-catenin signaling between wild-type MEFs and the G-alpha subunit null MEFs show the importance of the individual G-alpha subunits in limiting the activation of the β-catenin pathway in a time-dependent manner. In order to further understand the role of the individual alpha subunits, we used a transient genetic knockdown approach using specific short interfering RNAs (siRNAs) against G_q_, G_12_, and G_13_ subunits. Western blotting demonstrated over ∼70% knockdown efficiency of each G-alpha subunit (Fig. 5A).

The roles of G_q_, G_12_, and G_13_ in the regulation of active β-catenin were assessed individually. First, in contrast to the targeted null cells shown earlier (Fig. 3B), transient knockdown of individual G-alpha subunits did not affect basal β-catenin levels (Fig. 5). Upon stimulation, there were no significant differences observed with PMT and LiCl treatments in G_q_ knockdown cells compared to cells transfected with a nontargeting siRNA (Fig. 5B). In contrast, in G_13_ knockdown cells it was found that while PMT-treated cells showed no alterations in active β-catenin levels, treatment with LiCl showed a decrease in the levels of active β-catenin compared to the level for nontargeted controls (Fig. 5D). More importantly, PMT-mediated enhancement of LiCl signaling was lost in the absence of either G_12_ or G_13_ (Fig. 5C and D). These results suggest that short-term, transient knockdown of basal G_13_ negatively regulates the LiCl activation of β-catenin signaling and that G_12_ and G_13_ signaling is essential for regulation of LiCl-induced β-catenin signaling by PMT-activated G-proteins.

## DISCUSSION

**FIG 6:**
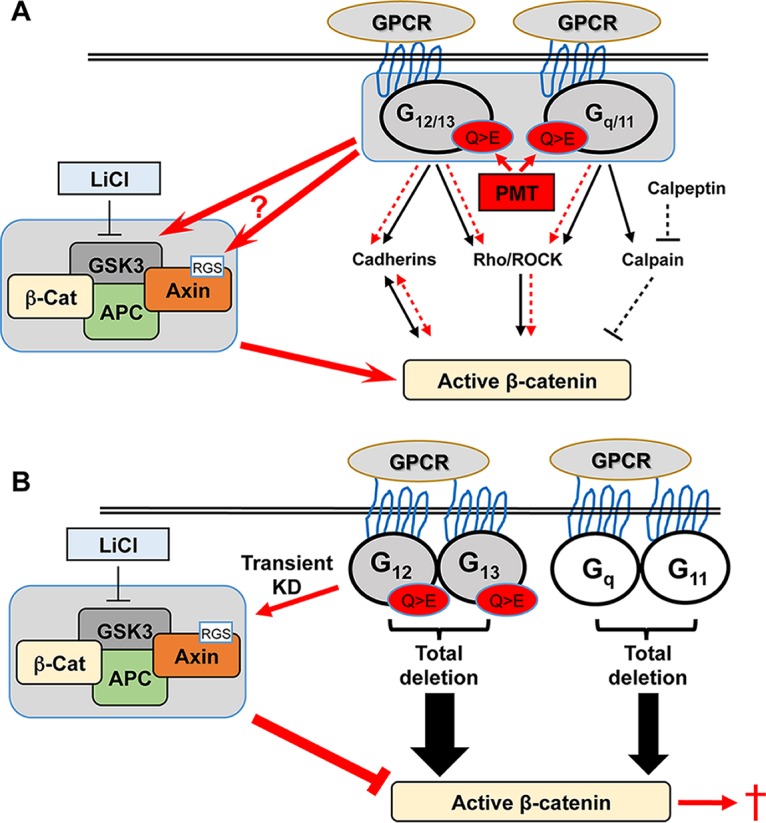
Model hypothesizing the potential regulation of β-catenin by G-alpha subunits and PMT. The classical G-protein targets for G_12/13_ (cadherins and Rho/ROCK) and G_q/11_ (Rho/ROCK and calpain) proteins and their stimulation of active β-catenin are indicated. (A) Regulation of β-catenin by PMT-modified G-alpha subunits. LiCl stimulates β-catenin signaling through inhibition of GSK. Proposed PMT-stimulated pathways are indicated in red. PMT-modified G-proteins (Q > E) enhance LiCl stimulation of active β-catenin levels (red arrows). This effect could be mediated through the known interactions between G-alpha subunits and either GSK3 or the RGS sequence in axin2. PMT might also affect G-protein interactions with cadherins and Rho/ROCK to induce β-catenin (red dashed arrows). Our data suggest that the G_q_-calpain pathway is not involved in PMT action (black dashed arrows). (B) Complete deletion of G-alpha subunits results in significant enhancement of β-catenin signaling in the absence of LiCl treatment (boldface black arrows), compromising cell viability (red dagger). PMT treatment (Q > E) of cells with transient knockdown (KD) of G_12_ and G_13_ (shaded) results in decreased β-catenin levels only via the Wnt destruction complex. See the text for details.

G-proteins are an important class of signal transduction proteins that play an essential role in development, maintaining cellular homeostasis, and in cancer (49, 50). Many studies have shown the regulation of their classical signaling pathways mediated by the specific G-alpha subunits, although there has been little discussion regarding any cross talk between these G-alpha subunits or with other pathways, such as β-catenin signaling. The regulation of β-catenin by G-proteins seems to be controversial. Some studies indicate that G-proteins positively regulate β-catenin, while others demonstrate their negative effect. We hypothesized here that this is due to differential effects of individual G-alpha subunits either in their basal state or in response to specific activators. Hence, to understand this potential cross talk better, we have dissected the role of basal and activated G-alpha subunits using genetic loss-of-function approaches to assess their effects on β-catenin signaling and have proposed a model to demonstrate these interactions (Fig. 6).

We have analyzed β-catenin signaling in MEFs and HEK293T cells using PMT as a unique tool to activate G-protein function in the absence of GPCR stimulation and have found that PMT enhanced LiCl-stimulated active β-catenin levels. The mechanisms underlying this synergistic effect are not completely understood, although previous studies have demonstrated both direct and indirect interactions between G-alpha subunits and components of the β-catenin signaling pathway. The clear membrane expression of active β-catenin following PMT and LiCl treatment suggests engagement with cadherins. Since G_12_ and G_13_ are known to interact with cadherins and release β-catenin from the cadherin complex (30, 51), our data suggest that PMT-activated G_12/13_ is responsible, at least in part, for the increase in active β-catenin. Alternatively, PMT could contribute to β-catenin activation through Rho/Rho kinase signaling. We and others have shown that PMT activates the Rho/Rho kinase pathway through both G_12/13_ and G_q/11_ (40, 52), and Rho kinase can stimulate β-catenin (53). Finally, active G_q_ has also been shown to inhibit GSK3β, leading to the accumulation of β-catenin (26). Thus, while G-alpha activation by PMT synergizes with LiCl to stimulate active β-catenin, it is difficult to identify the specific PMT targets that mediate this, as PMT activates all three G-alpha subunits in wild-type cells.

Cells harboring targeted G-alpha subunit knockouts showed upregulation of endogenous active β-catenin signaling in the absence of exogenous stimulation of either G-protein or Wnt/β-catenin signaling, and G_12/13_-deficient MEFs showed significantly higher basal β-catenin levels than G_q/11_^−/−^ MEFs. These findings suggest that endogenous G-alpha subunits are negative regulators of β-catenin signaling and, in particular, that G_12/13_ has greater effect in controlling β-catenin than G_q/11_. The precise mechanisms that regulate total versus active β-catenin protein levels are not clear at this time, as they are likely to differ between the different G-alpha subunits; thus, we focused our studies on active β-catenin. It is important to note, however, that changes in basal active β-catenin were observed only in the context of stable long-term inactivation of the G-alpha proteins using null MEFs, as transient knockdown of individual G-alpha subunits using siRNA did not affect basal active β-catenin levels. Thus, besides demonstrating clear regulation of active β-catenin by G-alpha proteins, our data suggest additionally that there are important differences in downstream signaling between transient versus long-term deletion of G-alpha subunits.

In MEFs deficient for either G_q/11_ or G_12/13_, which we showed have high endogenous β-catenin activity, additional PMT treatment did not affect β-catenin levels, and the enhancing effect of PMT on LiCl-induced β-catenin signaling was not observed. Longer treatment of G_12/13_^−/−^ but not G_q/11_^−/−^ MEFs with LiCl and/or PMT appeared to compromise cell viability. The reasons for this are not yet clear, but inhibition of GSK3β as well as high levels of β-catenin have been documented to stimulate apoptosis (54–56). Our observed upregulation of G_i_ in G_q/11_ knockout cells is consistent with what we (42) and others (17) have previously reported, and the levels of individual G-alpha subunits can also affect cell survival (57). Thus, the effects of altered levels of G-protein subunit activation, and indeed of GSK3β and β-catenin activity on cell survival, can vary widely in a cell context-dependent manner. Nevertheless, in the context of PMT and LiCl activation, our data clearly demonstrate that in the G-protein-null MEFs harboring constitutive loss of G-alpha subunits, activation of β-catenin is dependent upon functional G_12/13_ subunits. Interestingly, dissecting individual G-alpha subunit function by transient siRNA knockdown confirmed that G_12_ and G_13_, but not G_q_, play an essential role in agonist-mediated enhancement of β-catenin. Taken together, these data imply that G_12_ and G_13_ subunits protect cells from chronic activation of β-catenin that could cause pathological consequences, such as developmental defects and cancer (20, 58).

The mechanisms underlying G-alpha protein regulation of β-catenin are not clear. Our data suggest that one proposed mechanism involving G_q_-calpain interactions does not have a significant role. However, the observed increase in *Axin2* expression in both G_q/11_ and G_12/13_ null cells suggests that there is G-alpha subunit interaction with the destruction complex of Wnt signaling, as axin2 has a regulator of the G-protein signaling (RGS) site for G-alpha proteins, in particular for activated G_12_ (59). In some contexts, activated G_13_ and G_q_ have also been shown to bind to axin2 (35); thus, there is likely to be competition for axin2 binding among G-alpha subunits. Whether PMT treatment causes subcellular distribution of G-alpha proteins and binding to axin2 is not yet known, but this response would vary depending on which G-alpha proteins are present given the transient versus constitutive gain- or loss-of-function setting described here. Our data have pointed to a novel relationship between G-proteins and β-catenin signaling, namely, that the endogenous G-proteins serve to mediate a supervised activation of agonist-stimulated β-catenin signaling. However, where G-alpha subunit content has been altered, β-catenin signaling evades this control from G-proteins and is elevated. Hence, β-catenin regulation differs between the nonmodified cells and those with manipulated G-alpha subunits.

In summary, using the potent toxin PMT, which activates three of the four families of heterotrimeric G-proteins, and genetic deletions of G-alpha subunits, we have confirmed a link between G-alpha subunit activation and β-catenin signaling in the absence of canonical Wnt ligand stimulation. PMT provides an extremely useful tool to predict and test the effects of sustained activation of G-alpha subunits, which provides additional insights into the transient overexpression of constitutively activated subunits commonly reported. Indeed, our preliminary evidence suggests that PMT activates G-alpha subunits, especially G_12_, with kinetics different from those of other G-alpha subunits (C. Thompson, A. E. Grigoriadis, and A. J. Lax, unpublished data), and that long-term chronic treatment of cells with PMT continuously over months changes their endogenous G-alpha subunit content (A. Banu, A. E. Grigoriadis, and A. J. Lax, unpublished data). Our data also show that cells lacking G-proteins in the long term show potentially deleterious levels of β-catenin. However, short-term inhibition of G-proteins has no direct effect on β-catenin levels but was shown to alter agonist-mediated β-catenin signaling in a specific G-alpha-dependent way. Studying specific activated G-alpha subunits using PMT has revealed a specific role for G_12_ and G_13_ in cooperating with β-catenin signaling. Given the established roles of altered G_12/13_ proteins and β-catenin in tumorigenesis, it is tempting to speculate that PMT-induced G-protein fine-tuning of β-catenin signaling supports the procarcinogenic signature of chronic PMT exposure that we have previously hypothesized (60). Our findings suggest that the relative role of both basal and activated G-protein subunit levels will have to be understood before the full repertoire of their cellular function can be elucidated.

## MATERIALS AND METHODS

### Cell lines and preparation of whole-cell lysates.

Wild-type mouse embryonic fibroblasts (wild-type MEFs), G_q/11_^−/−^ MEFs, and G_12/13_^−/−^ MEFs were obtained from Stefan Offermanns, (Department of Pharmacology, Max Planck Institute for Heart and Lung Research, Bad Nauheim, Germany [47]). MEFs and HEK293T, MG63, and U2OS cells were cultured in Dulbecco’s modified Eagle medium supplemented with 10% fetal bovine serum (FBS), 50 units/ml of penicillin, 50 μg/ml of streptomycin, and l-glutamine, incubated at 37°C in a humidified atmosphere of 5% CO_2_. Equal numbers of cells were cultured in 6-well plates and grown until confluence. The cells were then incubated with the respective compounds as indicated in the figure legends for either 6 h or 24 h. After the respective treatment, cells were washed with ice-cold phosphate-buffered saline (PBS) and incubated with radioimmunoprecipitation assay (RIPA) buffer containing protease inhibitor cocktail (1:100 dilution) (P2714-1BTL; Sigma-Aldrich, Poole, UK) and phosphatase inhibitor cocktail (1:100 dilution) (40010; Active Motif, Carlsbad, CA) for 5 min on ice. Cells were removed using cell scrapers, and the whole-cell lysates were transferred to a microcentrifuge. The tubes were centrifuged at a relative centrifugal force of 14,000 for 15 min, and the supernatant was aliquoted and stored at −80°C. For anti-QE antibody preparation, hybridoma cells (a kind gift from Yasuhiko Horiguchi, Osaka University, Japan) were cultured in hybridoma-SFM medium supplemented with recombinant human interleukin-6 (IL-6) and purified as described previously (44).

### Western blotting.

The proteins were separated on 4 to 12% gradient gels and electrotransferred onto nitrocellulose membrane. The following antibodies were used: anti-active β-catenin antibody (8814S; New England Biolabs, Hitchin, UK) and total anti-β-catenin antibody (E5; Insight Biotechnology, Wembley, UK). All of the G-protein antibodies were purchased from Santa Cruz Biotechnology (Heidelberg, Germany): anti-Gα12 (sc- 409), anti-Gα13 (sc-410), anti-Gαq (sc-393), anti-Gα11 (sc-394), anti-Gαs (sc-823), anti-Gαi-1 (sc-391), anti-Gαi-2 (sc-7276), anti-Gαi-3 (sc-262), and anti-Gαo (sc-387). Bands were analyzed using the ChemiDoc MP imaging system from Bio-Rad. The band intensity was measured using Image Lab software (version 5.2.1). The band intensity of each protein was normalized against its β-actin housekeeping gene (61).

### Immunofluorescence.

Cells (5 × 10^4^ cells/ml) were cultured on 12-mm collagen-coated coverslips under standard conditions as described above and treated with the respective compounds. Cells were then fixed with 4% paraformaldehyde (PFA) for 1 h at room temperature (RT) and permeabilized with 0.2% Triton X-100 in PBS for 15 min at 4°C. They were then incubated with blocking buffer containing 1% bovine serum albumin in PBS for 30 min at RT. The primary anti-active β-catenin antibody and secondary anti-rabbit fluorescent antibodies were diluted in blocking buffer. The cells were incubated with the primary antibody (1:100) at 4°C overnight, washed with PBS, and incubated with secondary antibody (1:500) for 1 h at RT. The cells were washed with PBS three times and mounted using Vectashield mounting medium containing 4′,6-diamidino-2-phenylindole (Vector Laboratories, Peterborough, UK). The imaging was carried out using an ApoTome Zeiss fluorescence microscope, and the images were captured using an AxiCAm MRm Zeiss camera and processed on AxioVision Rel 4.8 software.

### G-protein knockdown.

siRNAs developed against GNAQ, GNA12, and GNA13 were purchased from GE Healthcare Dharmacon, Ltd. (Lafayette, CO): ON-TARGET plus human GNAQ (L-008562-00-0005), GNA12 (L-008435-00-0005), GNA13 (L-009948-00-0005), and nontargeting pool (D-001810-10-05). The transfection protocol was performed according to the manufacturer’s instructions. Briefly, HEK293T cells (2 × 10^4^ cells/well) were plated in a 24-well plate in medium containing no antibiotics and cultured overnight under standard conditions. Specific siRNAs and the transfection reagent were diluted in media without antibiotics in separate tubes. The contents of each tube were gently mixed and incubated for 5 min and then mixed and incubated for a further 20 min. Media were added into the above-described mix to obtain a final siRNA concentration of 25 nM. The cells were incubated with medium containing each siRNA for 72 h. Cells with each respective G-protein knockdown were analyzed by Western blotting for decreased expression and then used for further experiments. Significant knockdown of GNA11 expression was not achieved and was not pursued further.

### qPCR analysis.

For qPCR analysis, cells were cultured until confluence, and RNA was extracted using TRIzol (Invitrogen/ThermoFisher, Paisley, UK). Reverse transcription was performed using an RTnanoscript2 kit (Primer Design). The following primers were used: β-actin, 3′-GGCTGTATTCCCCTCCATCG-5′ and 5′-CCAGTTGGTAACAATGCCTGT-3′; axin2, 3′-TGACTCTCCTTCCAGATCCCA-5′ and 5′-TGCCCACACTAGGCTGACA-3′. The ΔΔ*C_T_* method (where *C_T_* is threshold cycle) was used for data analysis.

### Statistical analysis.

All experiments were repeated at least three independent times. One-way analysis of variance and Tukey’s test were used to assess the difference between treatments using GraphPad Prism software, version 7.
